# Changes in mortality of Polish residents in the early and late old age due to main causes of death from 2000 to 2019

**DOI:** 10.3389/fpubh.2023.1060028

**Published:** 2023-03-06

**Authors:** Monika Burzyńska, Małgorzata Pikala

**Affiliations:** Department of Epidemiology and Biostatistics, The Chair of Social and Preventive Medicine of the Medical University of Lodz, Lodz, Poland

**Keywords:** aging, causes of death, mortality trends, epidemiology, Poland

## Abstract

**Purpose:**

The aim of the study was to assess mortality trends in Poland between 2000 and 2019 in the early and late old age population (65–74 years and over 75 years).

**Methods:**

The work used data on all deaths of Polish residents aged over 65 years (*N* = 5,496,970). The analysis included the five most common major groups of causes of death: diseases of the circulatory system, malignant neoplasms, diseases of the respiratory system, diseases of the digestive system and external causes of mortality. The analysis of time trends has been carried out with the use of joinpoint models. The Annual Percentage Change (APC) for each segments of broken lines, the Average Annual Percentage Change (AAPC) for the whole study period (95% CI), and standardized death rates (SDRs) were calculated.

**Results:**

The percentage of deaths due to diseases of the circulatory system decreased in all the studied subgroups. Among malignant neoplasms, lung and bronchus cancers accounted for the largest percentage of deaths, for which the SDRs among men decreased, while those among women increased. In the early old age, the SDR value increased from 67.8 to 76.3 (AAPC = 0.6%, *p* > 0.05), while in the late old age group it increased from 112.1 to 155.2 (AAPC = 1.8%, *p* < 0.05). Among men, there was an upward trend for prostate cancer (AAPC = 0.4% in the early old age group and AAPC = 0.6% in the late old age group, *p* > 0.05) and a downward trend for stomach cancer (AAPC −3.2 and −2.7%, respectively, *p* < 0.05). Stomach cancer also showed a decreasing trend among women (AAPC −3.2 and −3.6%, *p* < 0.05). SDRs due to influenza and pneumonia were increasing. Increasing trends in mortality due to diseases of the digestive system in women and men in the early old age group have been observed in recent years, due to alcoholic liver disease. Among the external causes of mortality in the late old age group, the most common ones were falls.

**Conclusions:**

It is necessary to conduct further research that will allow to diagnose risk and health problems of the elderly subpopulation in order to meet the health burden of the aging society.

## Introduction

The process of population aging has demographic, economic, social and health dimensions. This is because the phenomenon is indirectly influenced by a number of factors, such as the level of affluence of the population, changes in the family model, professional activity of women, the quality of social and health care, education and government policies in the field of public health (1). Demographic forecasts predict that in 2050 the percentage of elderly people in the world will reach 16%. In EU countries, there will be only two people of working age for every person aged 65 years or over, while in Poland the share of people aged 65 years and over will be nearly 40%. The oldest age group, i.e., individuals over 85 years will constitute the largest group of people. The size of this group is expected to increase by more than 2.5 times as compared to 2020 (2). In Poland, at the end of 2020, the percentage of people aged 65 years and over was 23.8%, while the old-age dependency ratio, defined as the number of people aged 65 years and over per 100 people aged from 15 to 64 years, was 28.2. In view of the advancement of the population aging process, it is very important to analyse the health status of the elderly subpopulation (3, 4). Data on deaths are one of the most important sources of information on a population's health status in all age groups. Due to the fact that deaths have to be registered, they provide a database of complete information on the causes of mortality in societies around the world.

Based on the data from the Global Burden of Disease Study, between 1990 and 2017 as many as 12 million additional deaths worldwide were related to population aging. This accounted for 27.9% of all deaths, with the largest share attributed to ischemic heart disease (5).

The mortality structure and trends among the elderly reflect the mortality of the general population. In Poland, the predominant cause of death is cardiovascular disease, accounting for 42.6% of all deaths, with a ten-percentage-point decline since (6). The percentage of deaths from this cause in people aged over 65 years has also declined and is 41.1%. What is characteristic for the elderly is that the rate of deaths from cardiovascular disease among men only slightly exceeds that among women, while in younger age groups, mortality among men significantly exceeds that of women (7). The second most common cause of death in the general Polish population is cancer. The Health at a Glance 2021 report shows that the incidence of cancer in Poland has increased, which may indicate an improvement in early cancer diagnosis. However, the rate in Poland is still relatively low, reaching 267 per 100,000 population, with the average for OECD countries at 294, respectively. In contrast, the mortality rate from malignancies in Poland is one of the highest in OECD countries, at 228 deaths per 100,000 population, with an average of 191 per 100,000 (8). Cancer is also the second cause of death in people aged over 65 years (21.5%). Incidence trends in men in this age group showed an increase that continued until the mid-1990s, after which the phenomenon stabilized. In contrast, the elderly female population has seen an almost 1.6-fold increase in incidence over the past three decades. The majority of cancer deaths (75%) occur after the age of 60. The risk of dying from cancer increases with age, reaching a peak in the eighth and ninth decades of life (9). The third cause of death among the elderly, as in the general population, is respiratory diseases. They accounted for 6.5% of all deaths in 2020 and have shown an upward trend over recent years. Among those aged 65 years and older, respiratory diseases are almost twice as common a cause of death as among those under the age of 65 years. The next most common causes of death in the elderly population are digestive diseases (2.7%) and external causes of mortality (2.0%) (7).

The described changes in the age structure of the population determine the health profile of the society and the nature of challenges facing the health care system. More than 30% of patients using health care are affected by multi-morbidity, which is strongly related to age. The average annual cost of treating a patient over 65 years of age is almost three times higher than that of people in younger age groups. The demand for long-term care services is also growing. This gives rise to the need to look for solutions that will minimize the effects of the population aging i.a. by monitoring and forecasting the health needs of subpopulations in older age groups, separately from the population of younger people and those affected by premature mortality.

The aim of this study was to assess mortality trends in Poland between 2000 and 2019 in the early and late old age population.

## Materials and methods

The study used data on all deaths of Polish residents aged 65 years or more in the years 2000–2019 (*N* = 5,496,970). The database was based on death reports collected and made available for this study by the Department of Information of the Polish Central Statistical Office.

Mortality was analyzed in two age groups: early old age (65–74 years) and late old age (over 75 years). The analysis included the five most common major groups of causes of death: diseases of the circulatory system (according to the International Statistical Classification of Diseases and Health-Related Problems—Tenth Revision—ICD-10, coded as I00–I99), malignant neoplasms (C00–C97), diseases of the respiratory system (J00–J99), diseases of the digestive system (K00–K93) and external causes of mortality (V01–Y98). In each group, the most important causes of death were identified: ischemic heart diseases (I20–I25), cerebrovascular diseases (I60–I69), diseases of arteries, arterioles and capillaries (I70–I79), cancers of the lungs and bronchi (C34), stomach (C16), colorectal (C18–C20), breast (C50), prostate (C61), and pancreas (C25), chronic obstructive pulmonary disease (J44), influenza and pneumonia (J09–J18), alcoholic liver disease (K70), transport accidents (V01–V99), falls (W00–W19) and intentional self-harm (X60–X84).

The standardized death rates (SDRs) were calculated according to the following formula:


$$\begin{array}{l} \begin{array}{l} SDR=\frac{{\sum^{\text{​}}}_{i=1}^{N}\frac{ki}{pi}wi}{{\sum^{\text{​}}}_{i=1}^{N}wi} \end{array} \end{array}$$

where: k_i_ is the number of deaths in this i-age group, p_i_ is population size of this i-age group, w_i_ is the weight assigned to this i-age group, resulting from the distribution of the standard population, *N*—number of the age groups

The standardization procedure was performed using the direct method, in compliance with the European Standard Population, updated in 2012 (10). The Revised European Standard Population is the unweighted average of the individual populations of EU-27 plus EFTA countries in each five-year age band (with the exception of individuals under the age of five and the highest band, i.e., those aged over 85 years).

The analysis of time trends has been carried out with joinpoint models and Joinpoint Regression program, a statistical software package developed by the U.S. National Cancer Institute for the Surveillance, Epidemiology and End Results Program (11).

Joinpoint regression model is an advanced version of linear regression *y* = bx + a, where *b* is the slope coefficient, *a* is the y-intercept, y = ln(z), *z* is a measure evaluated in the study (SDR) and *x* is calendar year. Time trends were determined with the use of segments joining in joinpoints, where trend values significantly changed (*p* < 0.05). To confirm whether the changes were statistically significant, the Monte Carlo Permutation method was applied.

In addition, the authors also calculated the Annual Percentage Change (APC) for each segment of broken lines and the Average Annual Percentage Change (AAPC) for the whole study period with corresponding 95% confidence intervals (CI).

The Annual Percent Change is one of the ways to characterize trends in death rates over time and it was calculated according to the following formula:


$$\begin{array}{l} APC=100^{*}\left({\text{exp}}^{b}-1\right) \end{array}$$

where b is the slope coefficient.

With this approach, the death rates are assumed to change at a constant percentage of the rate of the previous year. For example, if the APC is 1%, and the rate is 50 per 100,000 in 2,000, the rate is 50 × 1.01 = 50.5 in 2001 and 50.5 × 1.01 = 51.005 in 2002. Rates that change at a constant percentage every year change linearly on a log scale.

The Average Annual Percent Change (AAPC) is a summary measure of the trend over a pre-specified fixed interval. It allows us to use a single number to describe the average APCs over a period of many years. It is valid even if the joinpoint model indicates that there were changes in trends during those years. It is computed as a weighted average of the APCs from the joinpoint model, with the weights equal to the length of the APC interval (12).


$$\begin{array}{l} AAPC=\left\{\exp\left(\frac{\sum w_{i}b_{i}}{\sum w_{i}}\right)-1\right\}\;\times \;100 \end{array}$$

where *bi* is the slope coefficient for each segment in the desired range of years and *wi* corresponds to the length of each segment in the range of years.

## Results

The most common major groups of causes of death among Polish residents aged over 65 years were the following: diseases of the circulatory system, malignant neoplasms, diseases of the respiratory system, diseases of the digestive system and external causes of mortality (Table 1).

**Table 1:** Percentage of deaths from the most common causes of death by gender in the groups aged 65–74 years and 75 years and older in 2000 and 2019.

**Sex**	**Men**			**Women**				
**Age group**	**65–74**		**75**+		**65–74**		**75**+	
**Year**	**2000**	**2019**	**2000**	**2019**	**2000**	**2019**	**2000**	**2019**
Diseases of the circulatory system (I00–I99) including:	45.63	35.19	56.22	44.87	48.40	30.82	63.34	51.84
Ischemic heart diseases (I20–I25)	18.01	11.91	16.27	13.05	15.71	9.13	15.62	12.62
Cerebrovascular diseases (I60–I69)	10.28	6.46	12.26	7.31	14.18	6.57	15.22	9.11
Diseases of arteries, arterioles and capillaries (I70–I79)	5.62	4.13	13.28	9.63	5.58	3.46	16.53	13.04
Malignant neoplasms (C00–C97) including:	31.96	33.97	18.90	22.31	29.83	41.84	12.35	14.33
Malignant neoplasm of bronchus and lung (C34)	11.89	10.99	4.77	4.82	3.51	10.24	1.02	1.77
Malignant neoplasm of stomach (C16)	2.58	1.91	1.75	1.26	1.82	1.33	0.94	0.59
Malignant neoplasm of colon (C18–C20)	2.76	4.11	2.01	3.37	3.25	4.07	1.67	2.08
Malignant neoplasm of breast (C50)	0.02	0.04	0.01	0.05	3.21	5.10	1.24	2.14
Malignant neoplasm of prostate (C61)	2.04	2.76	2.59	3.96	0	0	0	0
Malignant neoplasm of pancreas (C25)	1.15	1.63	0.66	0.75	1.66	2.61	0.73	0.87
Diseases of the respiratory system (J00–J99) including:	6.09	6.48	7.85	9.45	4.10	5.99	5.14	6.44
Chronic obstructive pulmonary disease (J44)	2.62	2.02	2.21	2.24	1.14	2.07	0.61	0.97
Influenza and pneumonia (J09–J18)	1.71	3.71	3.68	6.43	1.70	3.12	3.61	4.88
Diseases of the digestive system (K00–K93) including:	3.56	4.58	2.87	2.34	4.07	4.25	3.03	2.63
Alcoholic liver disease (K70) (K70–K74)	0.15	1.68	0.03	0.17	0.04	0.92	0	0.03
External causes of mortality (V01–Y98) including:	3.39	3.61	2.53	2.18	2.23	1.87	2.65	1.92
Transport accidents (V01–V99)	0.90	0.58	0.49	0.28	0.60	0.40	0.25	0.16
Falls (W00–W19)	0.57	0.80	1.05	1.13	0.66	0.53	1.76	1.35
Intentional self-harm (X60–X84)	0.67	0.84	0.34	0.34	0.30	0.29	0.09	0.05

The highest percentage of deaths in 2000 in both analyzed age groups (early old age and late old age cohorts), and in both gender groups were deaths caused by diseases of the circulatory system. Over the 20 years analyzed, this percentage decreased in all the subgroups studied. As a consequence, this led to a decrease in differences in death rates between diseases of the circulatory system and the second most common group of malignant neoplasms, while in the group of women in early old age in 2019, malignant neoplasms became the most frequent cause of death (Table 1).

**Figure 1:**
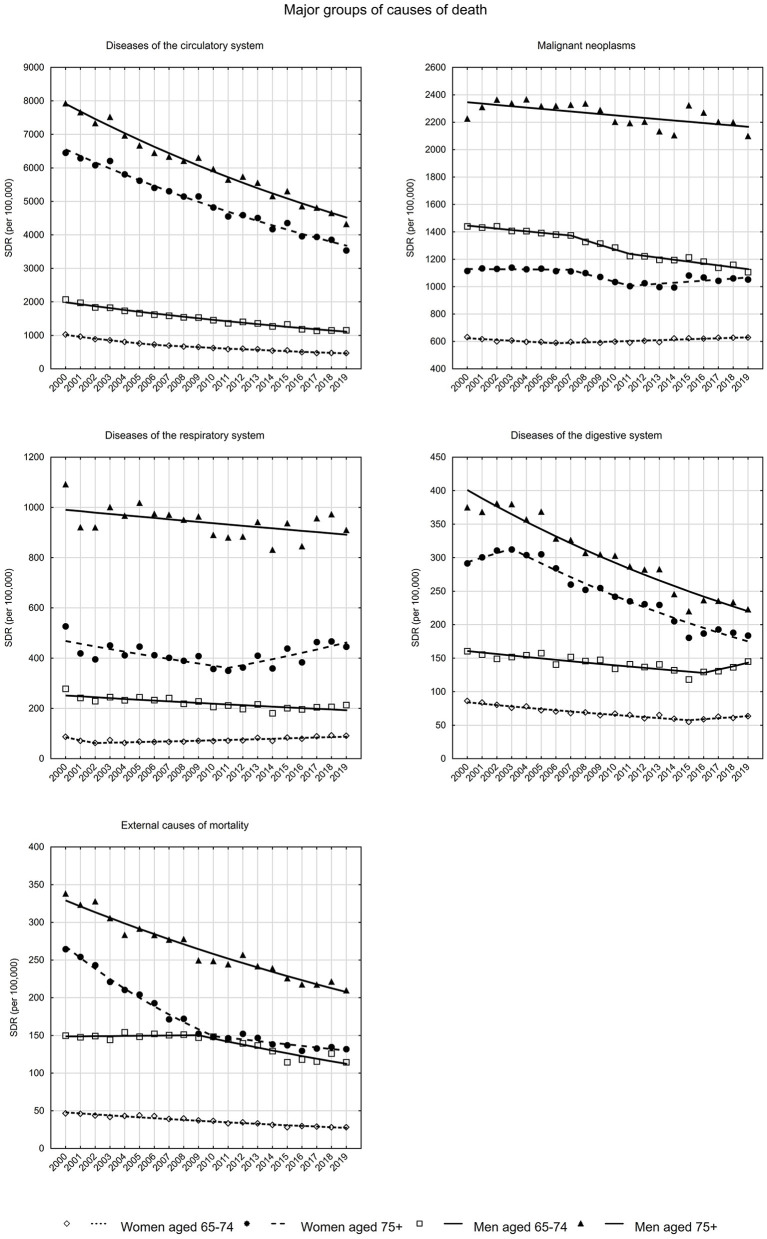
SDR trends in the Polish population aged 65–74 years and 75 years and older due to major groups of causes of death in the years 2000–2019.

The fastest percentage decline in deaths from cardiovascular diseases occurred in the group of women in early old age. The standardized death rate in this group decreased from 1027.3 in 2000 to 471.5 in 2019 (AAPC = −4.1%, *p* < 0.05) (Figure 1, Supplementary material 1, 2). In the other subgroups analyzed, AAPC was about −3.0% in 2019 (Supplementary material 2). In 2019, SDRs were 3,535.7 among women in late old age, 1,152.0 in the early old age male group, and 4,323.8 among men in late old age group (Supplementary material 1).

**Figure 2:**
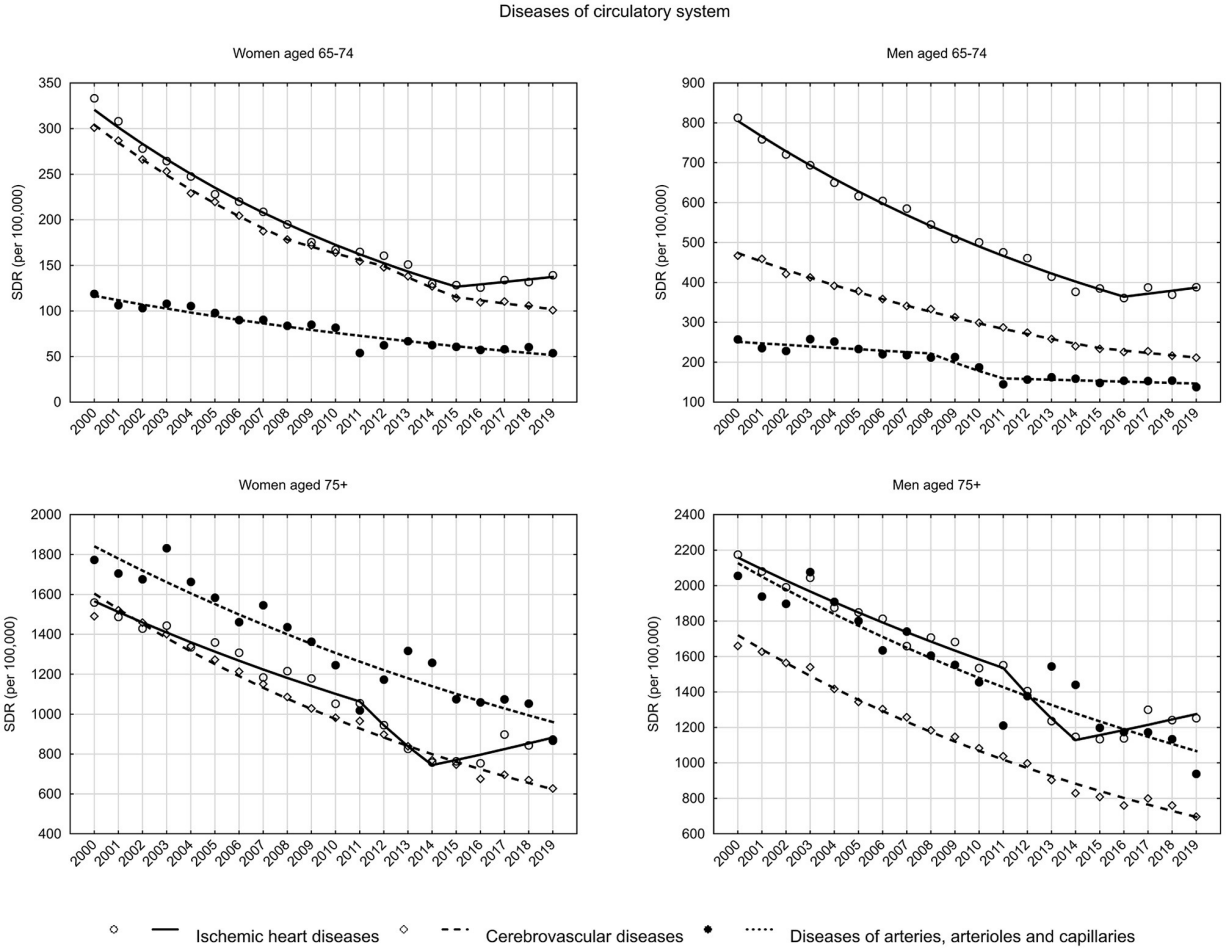
SDR trends in the Polish population aged 65–74 years and 75 years and older due to diseases of the circulatory system in the years 2000–2019.

Among cardiovascular diseases, ischemic heart diseases were the most common cause of death, except for women in late old age, where it was diseases of arteries, arterioles and capillaries (Figure 2). The third most common cause of death in the cardiovascular disease group involved cerebrovascular diseases. In each of the three aforementioned subgroups of causes of death among diseases of the circulatory system, in all the analyzed gender and age subgroups, decreasing trends were observed in the period between 2000 and 2019 (Supplementary material 2). A more detailed trend analysis, however, shows that SDRs due to ischemic heart disease have been increasing for a few years (Figure 2). In the early old age group of women and men, the upward trend began in 2015 and 2016, respectively, and was not statistically significant (APC = 2.1 and 2.0%). In the late old age group of women and men, the increase in SDR between 2014 and 2019 was statistically significant, i.e., APC was 3.5% in the female group and 2.5% in the male group (Supplementary material 2).

The share of malignant neoplasms among causes of death differed by gender and age. Among women in early late age, SDR was 631.4 in 2000 and decreased until 2006 at a rate of 1.0% (*p* < 0.05). After 2006, SDR began to increase (APC = 0.5%, *p* < 0.05). As a result, the SDR value in 2019 was 628.7 (Supplementary material 1, 2). There was a statistically insignificant decrease in SDR in the group of women in late old age between 2000 and 2011, and a statistically significant increase between 2011 and 2019 (APC = 0.7%). As a result, the SDR value decreased from 1,114.6 in 2000 to 1,004.1 in 2011, and then increased to 1,052.4 in 2019.

Among men, declining trends in SDR due to malignant neoplasms were observed in both age groups analyzed (Figure 2). A slightly faster decline occurred in the early old age group—from 1,440.1 in 2000 to 1,106.6 in 2019 (AAPC = −1.3%, *p* < 0.05). In the late old age group, the SDR values decreased from 2,226.5 in 2000 to 2,099.3 in 2019 (AAPC = −0.4%, *p* < 0.05) (Supplementary material 1, 2).

**Figure 3:**
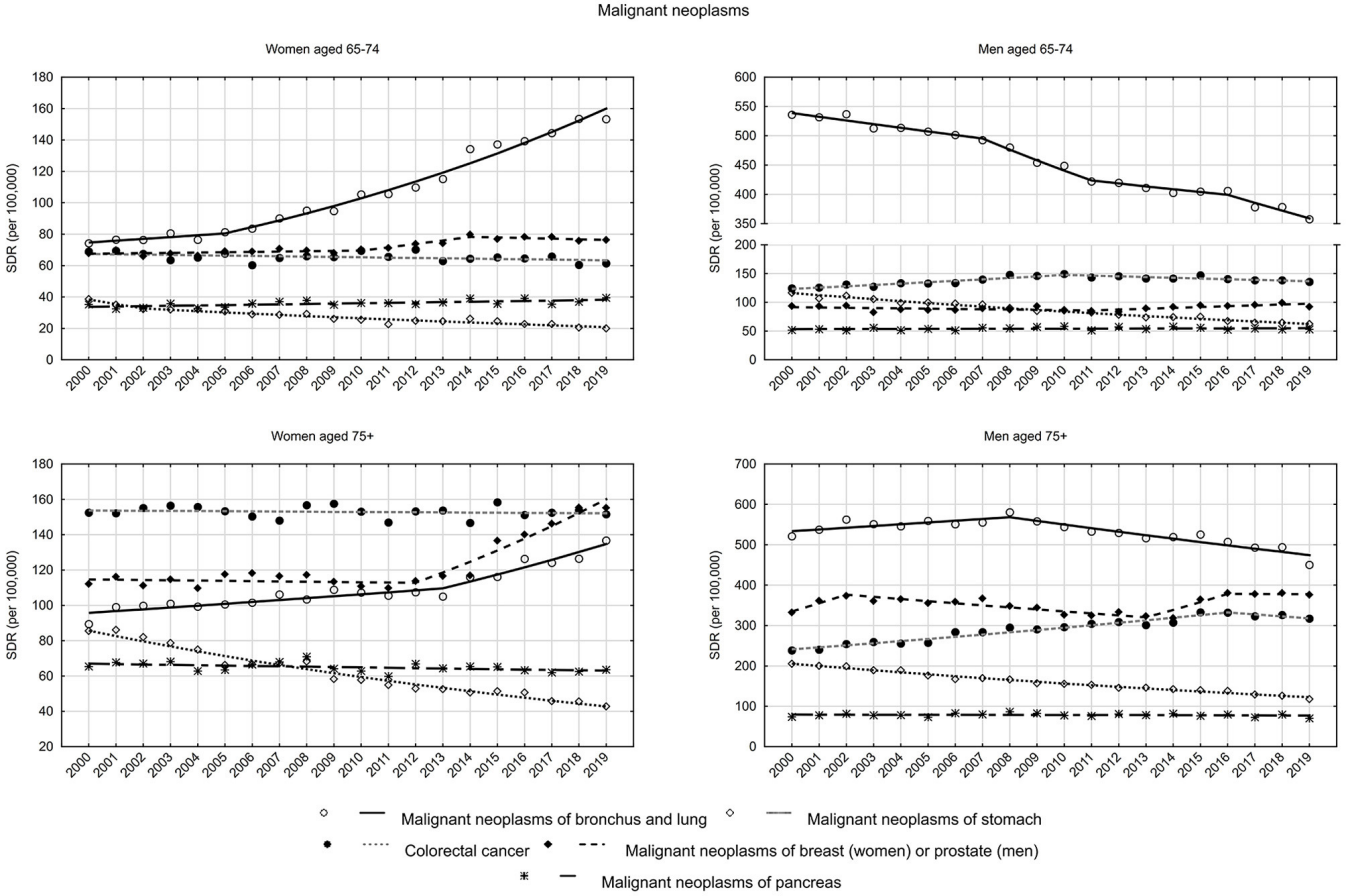
SDR trends in the Polish population aged 65–74 years and 75 years and older due to malignant neoplasms in the years 2000–2019.

Among malignancies, lung and bronchus cancer accounted for the largest share among causes of death, and while SDRs declined gradually in the male group, a continuous increase was observed in the female group (Figure 3). Among women in early old age, SDR increased from 74.2 in 2000 to 153.2 in 2019, with a small and statistically insignificant increase between 2000 and 2005 (APC = 1.5%, *p* > 0.05). After 2005, SDRs began to increase at a rapid rate of 5.0% (*p* < 0.05). Among women in late old age, there was an increase in SDR from 89.4 in 2000 to 105.0 in 2013 (APC = 1.1%, *p* < 0.05). Between 2013 and 2019, the increase accelerated to 3.5% (*p* < 0.05), with SDR reaching 136.7 in 2019. Among men in early old age, the SDR values decreased between 2000 and 2019 from 535.9 to 357.4 (AAPC = −2.1%, *p* < 0.05), and in late old age from 520.9 to 450.3 (AAPC = −0.6%, *p* < 0.05).

Among women, breast cancer was the second highest SDR cause of death among malignancies in the early old age group and the most common cancer causing death in the late old age group (Figure 3). In the early old age group, SDR increased in years between 2000 and 2014, and then began to decrease. As a result of these changes, the SDR value increased from 67.8 in 2000 to 76.3 in 2019 (AAPC = 0.6%, *p* > 0.05). In the group of women in late old age, a slight decrease in SDR between 2000 and 2012 (APC = −0.1%, *p* > 0.05) was followed by a rapid increase between 2012 and 2019 (APC = 5.1%, *p* < 0.05). As a result, the SDR value increased from 112.1 in 2000 to 155.2 in 2019 (AAPC = 1.8%, *p* < 0.05) (Supplementary material 1, 2).

Among men, prostate cancer and stomach cancer are the second and third causes of death among malignancies in the late old age group and the third and second causes in the early old age group. The opposite direction of trends was observed for these two cancers among men—increasing for prostate cancer (AAPC = 0.4% in the early old age group and AAPC = 0.6% in the late old age group, *p* > 0.05), and decreasing for stomach cancer (AAPC −3.2 and −2.7%, respectively, *p* < 0.05). There was also a downward trend observed among women in both analyzed age groups (AAPC −3.2 and −3.6%, respectively, *p* < 0.05) (Supplementary material 2).

Colorectal cancer mortality trends were stable among women in the early old age group (APC = −0.3%, *p* > 0.05) and in the late old age group (APC = −0.1%, *p* > 0.05). In the group of elderly men, SDRs due to colorectal cancer increased in the years 2000–2010 at a rate of 1.8% (*p* < 0.05), after 2010 they began to decrease at a rate of −0.8% (*p* < 0.05). In the group of elderly men, an increase in SDRs was observed in the years 2000–2016 (APC = 2.0%, *p* < 0.05) and a statistically insignificant decrease after 2016 (APC = −1.5%, *p* > 0.05) (Supplementary material 2).

Changes due to the fifth highest SDR cause of death among malignancies—pancreas cancer—were also analyzed. Increasing trends were observed in the early old age group (AAPC = 0.7%, *p* < 0.05 among women and AAPC = 0.2%, *p* > 0.05 among men) and decreasing trends in the late old age group (AAPC = −0.3%, *p* < 0.05 among women and AAPC = −0.1%, *p* > 0.05 among men) (Supplementary material 2).

**Figure 4:**
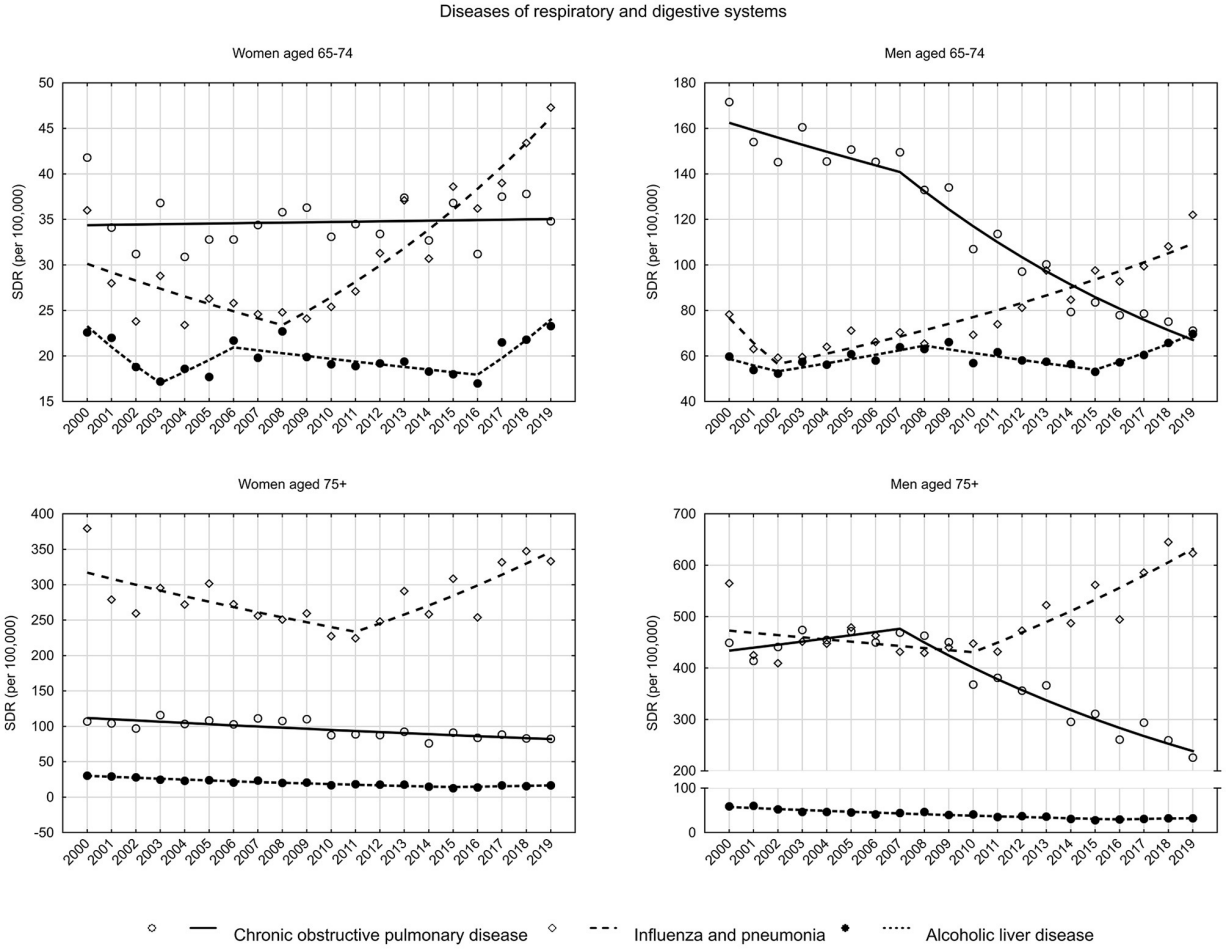
SDR trends in the Polish population aged 65–74 years and 75 years and older due to diseases of the respiratory and digestive system in the years 2000–2019.

Diseases of the respiratory system are becoming an increasingly common cause of death among women (Figure 1). In the early old age group, SDR increased from 63.1 to 90.9 between 2002 and 2009 (APC = 2.0%, *p* < 0.05), while in the late old age group, after a decline between 2000 and 2011 from 526.6 to 349.9 (APC = −2.3%, *p* < 0.05), an increase to a value of 445.5 in 2019 (APC = 3.1%, *p* < 0.05) began (Supplementary material 2). The increase in SDR due to diseases of the respiratory system was mainly influenced by influenza and pneumonia (Figure 4). Among early old age women, rates increased by 6.4% annually since 2008 (*p* < 0.05), while in the late old age group they increased by 5.1% per year since 2011 (*p* < 0.05). For the second most common chronic obstructive pulmonary disease, a stable SDR was observed throughout the analyzed period in the early old age group (APC = 0.1%, *p* > 0.05) and a decrease in the late old age group (APC = −1.6%, *p* < 0.05) (Supplementary material 1).

Among men, SDR values due to diseases of the respiratory system were decreasing. In the early old age group, SDRs decreased from 278.1 in 2000 to 213.4 in 2019 (APC = −1.4%, *p* < 0.05). In the late old age group, SDRs decreased from 1092.1 to 910.5 (APC = −0.5%, *p* < 0.05) (Supplementary material 1, 2). As in the female group, SDRs from influenza and pneumonia also increased in the male group. In the early old age group, the 2002–2019 APC was 4.0% (*p* < 0.05), while in the late old age group, the 2010–2019 APC was 4.3% (*p* < 0.05). In contrast, SDRs due to chronic obstructive pulmonary disease decreased, with an AAPC of −4.6% (*p* < 0.05) in the early old age group, and −3.1% (*p* < 0.05) in the late old age group (Supplementary material 2).

As for mortality from gastrointestinal diseases, in the last few years of the period studied, increasing trends in mortality were observed in the early old age group of women (as of 2015 APC = 2.5%, *p* > 0.05) and men (as of 2016 APC = 6.6%, *p* < 0.05) (Figure 1, Supplementary material 2). It is alcoholic liver disease, the most common cause of death in this disease group, that is responsible for these unfavorable trends. In 2016, in the group of women in early old age, APC was 10.2% (*p* < 0.05), while in 2015, in the group of men in early old age, it was 6.6% (*p* < 0.05). Moreover, SDRs due to alcoholic liver disease have also begun to increase in recent years in the late old age group. Among women, APC has been 3.7% (*p* > 0.05) since 2015, and among men it has been 3.3% (*p* > 0.05) since 2016 (Supplementary material 2).

The standardized death rates due to external causes decreased from 46.6 in 2000 to 28.1 in 2019 in the early old age group of women (AAPC = −2.9%, *p* < 0.05), whereas in the late old age group from 264.5 to 131.8 (AAPC = −3.7%, *p* < 0.05) (Figure 1, Supplementary material 1, 2).

**Figure 5:**
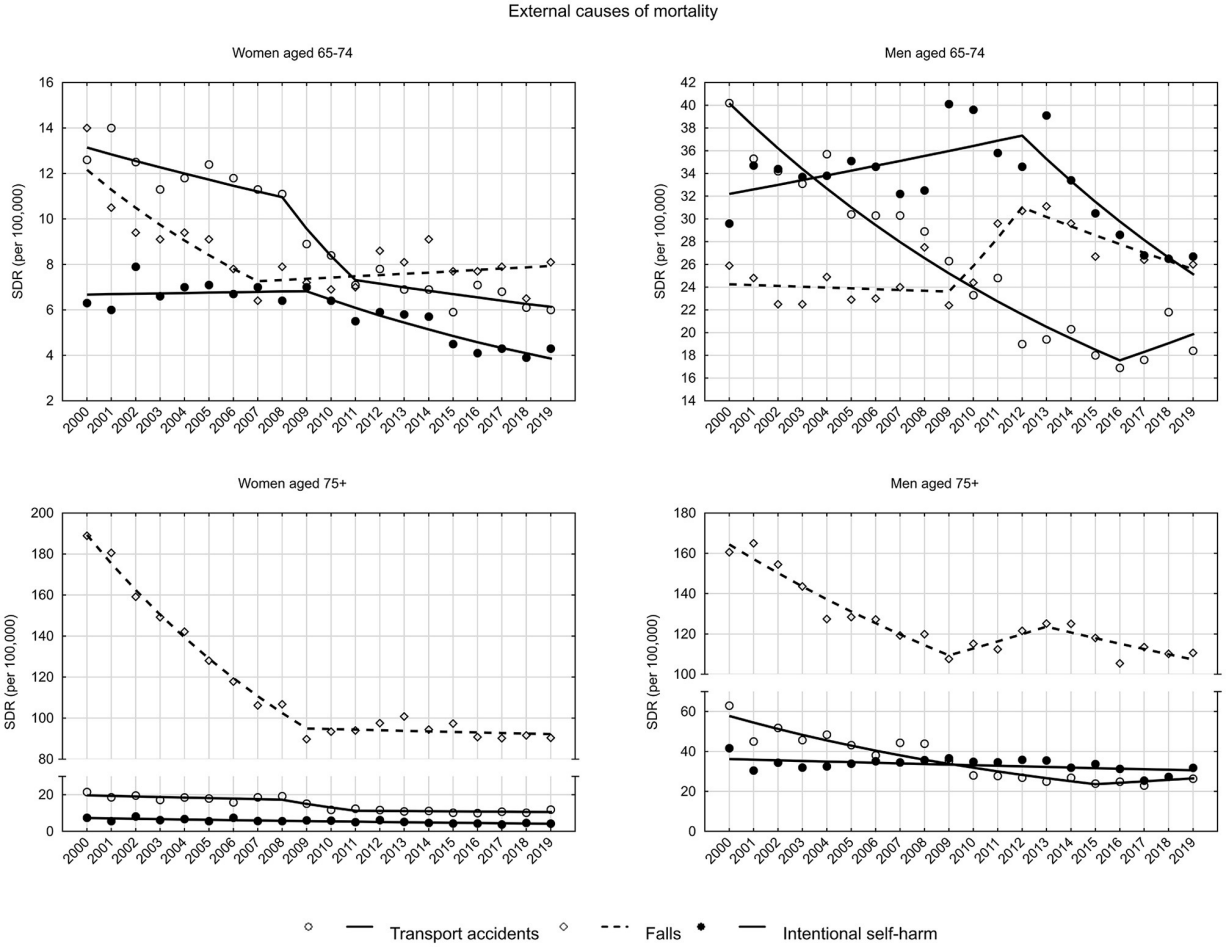
SDR trends in the Polish population aged 65–74 years and 75 years and older due to external causes in the years 2000–2019.

A decreasing trend in SDR due to transport accidents was observed in both age groups of women (in the early old age group AAPC = −3.9%, *p* < 0.05, in the late old age group AAPC = −3.2%, *p* > 0.05). Among men, a downward trend occurred between 2000 and 2016 in the early old age group (APC = −5.0%, *p* < 0.05) and between 2000 and 2015 in the late old age group (APC = −5.8%, *p* < 0.05). After this period, a statistically insignificant increase in SDR began (4.2 and 2.9%, respectively) (Figure 5, Supplementary material 2).

Among external causes of mortality in the late old age groups of both women and men, falls occurred most frequently. In the late old age group of women, a rapid decline in SDR between 2000 and 2009 (APC = −7.4%, *p* < 0.05) was followed by a period of stabilization (APC = −0.3%, *p* > 0.05). In the late old age group of men, there was a decreasing trend from 2000 to 2009 (APC = −4.4%, *p* < 0.05), then an increasing trend from 2009 to 2013 (APC = 3.1, *p* > 0.05) and again a decreasing trend from 2013 to 2019 (APC = −2.3%, *p* < 0.05) (Supplementary material 2).

As for suicides, downward trends were observed in the groups of early and late old age women (both groups AAPC = −2.8%, *p* < 0.05) as well as in the group of late old age of men (AAPC = −0.9%, *p* < 0.05). In the group of early old-age group men, SDRs increased between 2000 and 2012 at a rate of 1.2% (*p* < 0.05), after 2012 they began to decrease at a rate of −5.5% (*p* < 0.05) (Supplementary material 2). In both male age groups, SDRs due to suicide had higher values than those resulting from transport accidents in 2019 (Supplementary material 1).

## Discussion

Mortality in the elderly population is influenced by health and non-health determinants, particularly those of psychosocial nature. According to a study by J. S. House, these primarily include anti-health behaviors, such as poor dietary patterns, lack of physical activity, use of stimulants, lack of social contacts and support, stress, and inability to make decisions about one's own life (13). In order to explain the changes occurring in the mortality pattern of the elderly over the years, the causes of death have to be analyzed and their trends need to be assessed, which was accomplished in this study. The most common cause of death in the population of people aged 65 years and older were cardiovascular diseases. The risk of developing these conditions increases with age (14). According to data from the American Heart Association on Heart Disease and Stroke Statistics, the incidence of cardiovascular disease among patients aged 40–60 years is on average 35–40%, 60–80 years 75–78%, while among those aged over 80 years it exceeds 85%. At the same time, more than 80% of deaths in people aged over 65 years result from cardiovascular causes, and the same percentage of hospitalizations in this age group is due to this group of diseases (15). The Framingham Heart Study showed a significant relation of an increase in the incidence of coronary heart disease with age, in both men and women (16). In the Polish population, since 1990, favorable changes in overall mortality in all age groups have been observed, especially in relation to cardiovascular disease, which indicates the effectiveness of preventive measures taken, involving mainly those associated with lifestyle changes, including dietary improvements (17). In Poland, at the turn of 1989/1990, a socio-political transformation took place. Food was no longer subsidized after 1990; this caused big changes in relative prices. As a consequence, the structure of food consumed by Polish citizens changed substantially. For example, between 1989 and 2008 annual butter consumption decreased from 7 to 3.8 kg per head, and beef consumption fell by 75%. At the same time availability and consumption of fruits increased markedly (18). According to Bandosz et al. in the period between 1991 and 2005 about 54% of the deaths from coronary heart disease prevented or postponed were attributable to changes in risk factors and 37% to the increased use of evidence based treatments. Most (41% of the fall in men and 33% in women) were attributable to large decreases in mean cholesterol concentration (declining by 0.4 mmol/L). This fall in deaths concerns changes in mean cholesterol concentration related to diet only and was calculated by subtraction of drug related effects from total effect of mean cholesterol change. The effects of changes in smoking in men were observed also. The prevalence of smoking decreased by 15.7%, explaining about 15% of their fall in mortality. Mean systolic blood pressure fell by 2.7 mm Hg in men and by 5.2 mm Hg in women. After subtraction of the effects of treatments for hypertension, these falls in blood pressure explained about 29% of the decrease in mortality in women and 8% of the increase in deaths in men. Increased leisure time physical activity explained about 10% of the decrease in deaths. These gains were partially offset by about 1,810 additional deaths attributable to increases in BMI (−4 and −5% for men and women, respectively) and prevalence of diabetes (−1 and −8%, respectively) (6). However, these relatively favorable patterns weren't continued. As indicated by the results of the National Multicentre Health Survey WOBASZ II (2013–2014), the quality of Poles' eating habits, physical activity frequency, prevalence of obesity and overweight aren't satisfactory (19–21). This trend was confirmed in the present study. The favorable trend has been reversed for several years and the values of standardized mortality rates due to ischemic heart diseases (IHD) among the elderly, in all separate age groups in both sexes have increased. Studies show that the foundation of preventive and therapeutic measures among the elderly is regular physical activity (22, 23). In seniors with ischemic heart disease, appropriate physical exercise effectively slows the progression of the disease and lowers the risk of acute cardiac incidents, commonly referred to as myocardial infarctions, thus reducing the risk of death (24).

Although the percentage of people aged 60–69 who are physically active, meeting the dose of PA required for health recommendations, increased in Poland between 2014 and 2018 from 31.7 to 46.3%, age is the determining factor in these trends (25). In a study conducted in the Czech population, time spent on work-related and recreational physical activity decreased with age, while time spent in sedentary behaviors increased (26). A study by Biernat and Piatkowska shows that the problem of inactivity begins at the age of 50 years. On average, as many as 48.2% of Polish people aged 50–64 years do not follow the WHO recommendations (27). In the PolSenior2 study, age was also the most important determinant of declining physical activity (28). Also, a 2018 report issued by the Central Statistical Office (CSO) confirms that older Polish residents are less active as compared to younger individuals (25.1 vs. 46.4% on average) (4). This results in the fact the share of physically active people aged 65 years or more remains insufficient and much lower than in other EU countries (29). Considering how important regular physical activity is for prevention of chronic non-communicable diseases, including ischemic heart disease, it may be assumed that it is this factor that plays a significant role in the unfavorable mortality trends due to IHD, offsetting the impact of favorable changes in other lifestyle components (30). The worsening trend in mortality in the elderly population from this cause observed in recent years can also be attributed to the significant increase in the prevalence of obesity, diabetes and metabolic syndrome, the co-presence of which significantly increases the risk of death from IHD (31). Another important health problem whose incidence is closely correlated with age is cancer (32). In 2019, nearly 50% of cancer deaths were reported in the subpopulation of people aged 65 years and older (33). According to the authors' results, malignant neoplasms were, as in the general population, the second cause of death in the population aged over 65 years. However, mortality trends have been inconclusive both in general and with regard to individual malignancies. An overall increase in standardized mortality rates from malignant neoplasms in the female group has been observed in recent years, with a concomitant decline in men in both age groups. Studies of cancer mortality trends from 1970 to 2015 conducted in 11 countries around the world confirm these unfavorable Polish trends in comparison with other countries, for which mortality patterns over the past few decades have varied, however, have been more optimistic. They also confirm a significantly faster reduction in mortality levels for men than for women (34). It is also worth referring to trends in mortality due to specific types of cancer. The analysis showed that bronchus and lung cancers accounted for the largest share among causes of death, and while SDRs declined steadily in the male group, a steady upward tendency was observed in the female group. Similar trends have been observed in most European countries, with decreases in incidence and mortality from lung cancer since 2000. A significant decrease has also been recorded in North America and the United Kingdom (35). This is due to the decline in smoking prevalence among generations of men. In comparison, among women, smoking prevalence increased in the US and UK after World War II, and in the 1970's in most other countries as well, i.e., in the generation born between the 1930's and 1950's (36). Moreover, middle-aged and older men were more likely to quit smoking than women. It should also be remembered that lung cancer risk factors translate into morbidity and resulting mortality with a lag of up to even more than 20 years. Therefore, the incidence and mortality of lung cancer in women aged over 65 years in various regions of the world continues to increase (37). The positive change in the mortality trend among Polish men is also a consequence of the declining prevalence of smoking in all age groups. In contrast to women, where active smoking varies greatly by cohort effect (period of birth in calendar time). The highest smoking rate was observed in the generation of women born between 1940 and 1960. In the population of women born after 1960, smoking prevalence has halved and is now 20.0–25.0%. Exposure to the carcinogens of tobacco smoke, after taking into account the 20-year latency period, accurately explains the trends of lung cancers in older women in Poland, while the observed cohort effect means that the incidence of the disease, and the resulting mortality, still shows an upward trend that will continue for some time in the future (38). Prostate cancer is also listed among malignancies strongly associated with smoking. It is estimated that by 2040, mortality related to prostate cancer in the general population will double as compared to 2018, reaching 379,005 deaths worldwide. The highest mortality rate will occur in Africa (+124.4%) and Asia (116.7%), while the lowest in Europe (+58.3%) (39). Currently, prostate cancer mortality trends are not clear-cut and show global territorial variations. In the population of older men, after an increase in mortality occurring until the 1990's, significant declines in SDR from this cause are observed in North American countries, Argentina, Australia and most European countries, except Poland and Russia. The most favorable changes are recorded in Japan. The rates declined between 2002 and 2012 (9.8%), reaching 61.6/100,000 men in 2012 (40). Since 2015, the number of deaths from prostate cancer in EU countries has dropped by an average of 7%, which is attributed to improved treatment and better diagnosis (41). Unfortunately, Poland is the only country to which this indicator does not apply, as for the past 5 years there has been a steady increase in mortality due to late diagnosis, among others. A significant number of patients still remain undiagnosed. According to the National Cancer Registry in Poland, the annual rate of increase in incidence is estimated at 2.5%, however, the risk of incidence increases markedly after the age of 50, and after the age of 80 the cancer is found in almost 80% of men (42), which explains the increasing trend for prostate cancer observed in our study in men aged over 65 years, in early and late old age.

Negative trends have also been observed in the early old age group for pancreas cancer in both men and women. Similar trends have also been observed in younger age groups in the rest of the world. However, the reason for these unfavorable tendencies remains largely unexplained (43).

Beginning in the 1990s, as a result of the introduction of screening tests, early diagnosis and improved treatment, favorable global trends in breast cancer mortality among older women have been observed (44). At the same time, however, upward trends have been observed in Asian countries. In Japan, the rate of increase in the mortality rate between 1970 and 2015 was 2.2. An upward trend in the mortality rate was also observed in Russia (by 10.3%), as well as in Poland (45), which was confirmed in our study in the group of women in late old age. At the time they entered the age of increased risk of developing the disease, preventive measures leading to early detection and high survival rates were not yet as widespread as they are today. The reduction in mortality from breast cancer is influenced by population-based screening programs, participation in which increases the chance of rapid diagnosis and effective treatment. In Poland, the breast cancer screening program began in 2006. By comparison, in the United States it was introduced 20 years earlier. Thus, the current epidemiological picture does not yet show clear unidirectional changes resulting from the participation of Polish women in this program (46).

Our study also analyzed trends in mortality from stomach cancer, showing a decrease in all four age and gender groups. The absolute incidence of stomach cancer has been growing slightly worldwide as a result of an increase in the size and average age of some populations. However, in most countries, the incidence of stomach cancer has declined by about 75% over the past 50 years. Mortality from this cause in all age groups has also declined. In the United States, the mortality rate has dropped from 37 to 6 per 100,000 people. Japan, too, has seen a decline of almost 40%. Studies suggest that this is due to early detection of stomach cancer, changes in dietary habits, increased levels of hygiene, reduced tobacco smoking among men and, most importantly, a decrease in the incidence of *Helicobacter pylori* infection (47). A study by Ostrowski et al. found an ~30% lower prevalence of *Helicobacter pylori* infection in Poland as compared to studies conducted 15 years ago (48).

Our study showed that diseases of the respiratory system were an increasingly common cause of death in the group of women aged over 65 years during the period analyzed, mainly due to an increase in mortality from influenza and pneumonia. Although, in general, decreasing trends in mortality from diseases of the respiratory system were observed in the group of men, the values of standardized mortality rates for influenza and pneumonia were increasing. This unfavorable trend observed in Poland is attributed to the unsatisfactory level of vaccination against influenza and the change in its etiological factor. Year by year, the disease is increasingly caused by the A strain (78% of cases in 2019), which is responsible for the severe course of the disease and increases the risk of complications such as pneumonia, exacerbation of chronic disease or myocarditis, which become the ultimate cause of death in the elderly. The likelihood of death, as well as severe flu complications requiring hospitalization, increase nearly threefold in people aged over 65 years. Of critical importance in protecting the safety of the elderly is immunization (49). According to the WHO recommendations, influenza vaccination among the elderly in the WHO European Region should be implemented at 75% of the vaccination status in this age group. Data on the influenza vaccination status of the elderly in EU countries show that it is about 44% on average, but varies from country to country (above 75% in the Netherlands, 43% in Denmark, 68% in the UK, 57.6 in Ireland, 10% in Poland, 6.9% in Latvia and 4.8% in Estonia) (50). According to data from the National Institute of Public Health - National Institute of Hygiene, the level of influenza vaccination in the population aged over 65 years fluctuated between 2009 and 2018, and unfortunately shows a downward trend from 11.35% in 2009 to the lowest value in 2016–6.87%. In 2018, the percentage of seniors vaccinated against influenza was 8.31% (51). In Poland, in response to these unfavorable trends, a 50% reimbursement of influenza vaccination for people aged over 65 years was introduced in 2018, while in 2020 it was extended to include free vaccination for people aged over 75 years. Interest in this form of prevention, especially in the senior population, increased during the COVID-19 pandemic, which gives hope that social awareness of the role of immunization in the fight against infectious diseases will gradually improve.

Another group of diseases whose incidence increases with age are those of the digestive system. The most common cause of deaths analyzed, accounting for adverse mortality trends in this group, was alcoholic liver disease (ALD). Mortality from this cause continues to be an important public health problem. Globally, alcohol accounts for 7.6% of deaths in men and 4.0% of deaths in women. It is Europe that consumes the most-−10.9 liters per person per year. For the past 25 years or so, average alcohol consumption in Central and Eastern Europe has remained stable, in Western and Southern Europe it has decreased, while in the UK and Finland it has increased (52). According to data from the National Agency for Solving Alcohol Problems, consumption of 100% alcohol per capita in Poland, despite isolated declines (related to higher rates excise tax introduced in 2009 and 2014, among others) has shown an upward trend. Currently, recorded levels are significantly higher than in the early 1990s (53). Deaths related to excessive alcohol consumption are most often due to cardiovascular diseases, transport accidents and alcoholic liver disease. In the European Union, 41% of liver disease deaths have alcohol-consumption background, and in 46% the cause is unknown, however, it is likely to be very often related to alcohol as well. The social and economic costs of excessive alcohol consumption are enormous, hence ALD remains a very important civilization challenge (54).

Unfavorable trends regarding total deaths related to alcohol consumption were demonstrated in a study by Zatoński et al. Although the highest mortality rates were recorded in the group of Polish residents aged 45–64 years, the rate of increase in the years 2002–2017 was the fastest in the population of people aged over 65 years, both among men and women (AAPCs were 8.5 and 12.2, respectively). These unfavorable trends can be fully linked to the weakening of alcohol control measures in Poland. At the same time, alcohol-related mortality has decreased in countries such as Russia and Lithuania, where new, stricter methods for controlling alcohol consumption in the population have been introduced (55). In the United States, between 1999 and 2019, there was also a statistically significant increase in mortality from alcoholic cirrhosis in each of the 10-year age groups analyzed (25–85 years and older). The largest increase also occurred in early old age—in individuals aged 65 to 74 years—and the differences between men and women in this group gradually disappeared to the disadvantage of women (56).

A study on drinking culture among people aged 60–64 years in Poland was the Standardized European Alcohol Survey (RARHA SEAS). The subpopulation covered by it included retirees from the so-called “baby boomers” generation, those born between 1945 and 1964. This is the generation of the post-war demographic peak coinciding with widespread shortages of consumer goods. As they entered adulthood, echoes of the cultural revolution of the 1960's reached Poland and influenced the generation of Polish baby boomers, including in terms of alcohol consumption. Statistics from the 1970's and early 1980's show very high levels of alcohol consumption, which may indicate a risky drinking pattern for many people of this generation (57). Considering the fact that ALD is diagnosed with a long delay (58), after many years of alcohol dependence, this may explain the unfavorable trends related to alcoholic liver disease among those included in this study. Undoubtedly, these alarming trends in mortality from this cause represent a health challenge aimed at reducing alcohol consumption in Polish society (59), especially in the era of the COVID-19 pandemic, which had a negative impact on its patterns (60).

One of the most important public health problems globally are injuries, resulting from external causes, mainly transport accidents, self-harm and suicide attempts (61). Data on the incidence of hospital treatment in Europe indicate that the incidence of injuries is bimodal—clearly increasing among both young people and those aged over 60 years, however, with a change in the hierarchy of their causes (62). In the old population, the share of falls increases, accounting for nearly 69% of outpatient and inpatient treatment for all external causes in EU countries vs. 41% in the under-65 group. In contrast, the share of transport accidents decreases with age (6 vs. 10%, respectively). Despite the relatively favorable trends in mortality from falls and transport accidents shown in this study, the risk of death for Polish seniors from each of these causes is higher than the EU average by 23 and 53%, respectively (63).

A significant problem in the group of external causes of death among the elderly is suicide (64). The study including a group of people aged 65 years and more shows that the average suicide rate among older men in Europe is significantly higher than the average among older women (65), as also shown in a study by Law *et al*. conducted in Australia (66), as well as our own study. At the same time, the mental health of the Polish population is deteriorating. Between 1997 and 2010, the number of people suffering from mental disorders increased (67). This is particularly worrisome in old age, when deteriorating health with age, multi-morbidity and polypharmacy increase the risk of mental disorders predisposing to a suicidal act (68). A factor that increases this risk is the moment when people decide to retire. The inability to fulfill oneself at work, as well as deterioration of the financial condition often associated with retirement, increase the risk of depression, from which the risk of death increases with the severity of symptoms (69, 70). Studies also show that seniors do not report their suicidal intentions and are more likely to make attempts in conditions where intervention is not possible, which is especially true for men (71). Our own study showed favorable trends in mortality from suicide in all groups except for men in early old age. This calls for special observation in order to take appropriate preventive measures in the male population, for whom SDRs due to suicide had higher values in 2019 than those due to transport accidents in both early and late old age.

The results of the Survey of Health, Aging and Retirement in Europe, carried out in 2017 in 27 European countries, indicate that the health status of Polish people aged 50 years and older is significantly worse than that of populations of other countries, such as Sweden, Greece, or Spain. The survey also proves a higher risk of chronic diseases and a faster rate of their increase with age (72).

Our study had some limitations. Quality of the analyses performed on the mortality statistics depend on the completeness and accuracy of the information contained in the death certificate and the proper and precise description of the cause of death. Poland is a country with 100% completeness of death registration. In order to standardize death causes, which are subject to further statistical analyses, it was determined that the doctor who pronounces death is responsible for filling in the death card, into which he or she puts the primary, secondary and direct death cause, whereas qualified teams of doctors are responsible for coding death causes according to the ICD-10 classification. The data relating to 2000 shows that the cause of 24.8% of deaths were inaccurately described. In 2015 this percentage was the highest and amounted to 31.2%, after which it steadily decreased and in 2019 it amounted to 27.4%.In the majority of cases garbage codes concerned deaths due to cardiovascular diseases. Significantly fewer incorrect codes number concerns other causes of death (73).

However, from the perspective of public health, it is so important to assess the health burden of the elderly population (74). It will allow for taking appropriate measures aimed at improving the quality of life and gradually increasing the years lived in health.

## Conclusions

The percentage of deaths due to diseases of the circulatory system decreased in the studied subgroups but this problem still remains the greatest health risk in the elderly population, primarily due to ischemic heart disease for which growing trends were observed in recent years of analyzed period. Among malignant neoplasms, lung and bronchus cancer accounted for the largest percentage of deaths, for which the analyzed trends were growing among women and decreasing in male group. Unfavorable trends in mortality due to prostate cancer in the group of men in the early old age and due to breast cancer in the group of women in the late old age were observed. Mortality due to stomach cancer was steadily decreasing in all analyzed subgroups. Diseases of the respiratory system are becoming an increasingly common cause of death among women, mainly due to influenza and pneumonia. Increasing trends in mortality due to diseases of the digestive system in women and men in the early old age group have been observed in recent years, due to alcoholic liver disease—the most common cause of death in this disease group. Downward trends of mortality due to external causes, mainly according to suicides, were observed in both gender groups. It is necessary to conduct further research that will allow to diagnose risk and health problems of the elderly subpopulation in order to meet the health burden of the aging society.

## Institutional review board statement

The study was conducted according to the guidelines of the Declaration of Helsinki, and approved by the Bioethics Committee of the Medical University of Lodz on 22 May 2012 No. RNN/422/12/KB.

## Data availability statement

The raw data supporting the conclusions of this article will be made available by the authors, without undue reservation.

## Ethics statement

The studies involving human participants were reviewed and approved by Bioethics Committee of the Medical University of Lodz, No. RNN/422/12/KB. Written informed consent for participation was not required for this study in accordance with the national legislation and the institutional requirements.

## Author contributions

MB contributed to study design and writing the article. MP conducted the statistical analysis and interpreted the data. All authors participated in the critical revision of the article and approved the final article.
