# Supplementary material for: Changes in mortality of Polish residents in the early and late old age due to main causes of death from 2000 to 2019
Source: Front Public Health. 2023 Mar 6;11:1060028. doi: 10.3389/fpubh.2023.1060028 (PMC10025537; doi:10.3389/fpubh.2023.1060028)
Supplement: Supplementary file 1 [file Table_1.pdf]

Supplement 1. SDR values related to the most common causes of deaths in the years 2000-2019.

Women 65-74

Causes of death	2000	2001	2002	2003	2004	2005	2006	2007	2008	2009	2010	2011	2012	2013	2014	2015	2016	2017	2018	2019
Diseases of the circulatory system (I00-I99) including:	1027.3	960.6	885.4	851.7	803.3	757.6	725.2	694.6	661.9	651.2	624.0	584.5	601.2	584.3	541.5	552.0	495.0	473.0	474.7	471.5
Ischemic heart diseases (I20-I25)	333.3	308.1	278.1	264.4	247.5	228.1	219.9	208.7	194.9	175.4	167.3	164.7	160.7	151.0	129.9	128.4	125.7	134.0	131.9	139.2
Cerebrovascular diseases (I60-I69)	300.9	286.7	266.0	253.1	229.0	219.6	204.4	187.4	178.3	172.0	164.1	154.5	147.9	138.1	127.0	114.2	109.5	110.3	105.8	100.8
Diseases of arteries, arterioles and capillaries (I70-I79)	118.7	106.4	103.1	107.9	105.3	97.9	90.1	90.3	83.8	85.0	81.6	53.9	62.5	66.8	62.6	60.7	57.3	58.0	60.3	53.7
Malignant neoplasms (C00-C97) including:	631.4	616.8	600.5	606.6	597.0	596.2	589.9	597.1	604.6	590.0	598.4	590.4	603.9	594.6	622.1	623.4	619.9	627.4	627.4	628.7
Malignant neoplasm of bronchus and lung (C34)	74.2	76.5	76.3	80.5	76.4	81.3	83.5	90.1	95.0	94.7	105.3	105.5	109.8	115.2	134.2	137.2	139.3	144.4	153.4	153.2
Malignant neoplasm of stomach (C16)	38.6	35.1	32.3	32.0	32.1	30.8	28.9	28.7	29.2	26.0	25.5	22.6	24.8	24.5	26.1	24.5	22.8	22.8	20.5	19.9
Colorectal cancer (C18-C20)	68.9	69.4	67.5	63.4	65.1	67.6	60.3	64.9	65.9	65.4	69.4	65.6	70.2	62.9	64.4	65.2	64.6	65.8	60.5	61.4
Malignant neoplasm of breast (C50)	67.8	69.8	66.0	67.6	66.4	69.2	68.9	70.7	69.6	68.0	70.3	71.2	73.8	74.2	79.8	76.9	78.3	78.3	75.8	76.3
Malignant neoplasm of pancreas (C25)	35.2	32.4	33.1	35.8	32.8	33.6	35.8	37.0	37.8	35.1	36.2	36.1	35.5	36.6	39.1	35.7	39.2	35.5	36.9	39.4
Diseases of the respiratory system (J00-J99) including:	86.9	70.8	63.1	74.0	62.8	67.9	66.5	66.9	67.2	71.3	69.6	71.5	72.1	82.8	70.4	84.0	79.0	89.2	92.6	90.9
Chronic obstructive pulmonary disease (J44)	41.8	34.1	31.2	36.8	30.9	32.8	32.8	34.4	35.8	36.3	33.1	34.5	33.4	37.4	32.7	36.8	31.2	37.5	37.8	34.8
Influenza and pneumonia (J09-J18)	36.0	28.0	23.8	28.8	23.4	26.3	25.8	24.6	24.8	24.1	25.4	27.1	31.3	37.1	30.7	38.6	36.2	39.0	43.4	47.3
Diseases of the digestive system (K00-K93) including:	86.2	83.8	80.2	75.8	78.0	72.1	70.4	68.0	69.0	64.8	67.1	65.1	60.2	65.1	59.6	54.7	58.8	62.6	60.7	63.4
Alcoholic liver disease (K70)	22.6	22.0	18.8	17.2	18.6	17.7	21.7	19.8	22.7	19.9	19.1	18.9	19.2	19.4	18.3	18.0	17.0	21.5	21.8	23.3
External causes of mortality (V01-Y98) including:	46.6	46.1	43.7	41.8	43.1	43.9	42.8	39.2	39.8	37.2	36.5	33.3	34.7	33.3	31.2	28.2	29.6	29.0	28.1	28.1
Transport accidents (V01-V99)	12.6	14.0	12.5	11.3	11.8	12.4	11.8	11.3	11.1	8.9	8.4	7.1	7.8	6.9	6.9	5.9	7.1	6.8	6.1	6.0
Falls (W00-W19)	14.0	10.5	9.4	9.1	9.4	9.1	7.8	6.4	7.9	7.2	6.9	7.0	8.6	8.1	9.1	7.7	7.7	7.9	6.5	8.1
Intentional self-harm (X60-X84)	6.3	6.0	7.9	6.6	7.0	7.1	6.7	7.0	6.4	7.0	6.4	5.5	5.9	5.8	5.7	4.5	4.1	4.3	3.9	4.3

Women 75+

Causes of death	2000	2001	2002	2003	2004	2005	2006	2007	2008	2009	2010	2011	2012	2013	2014	2015	2016	2017	2018	2019
Diseases of the circulatory system (I00-I99) including:	6452.0	6289.5	6078.7	6205.5	5809.0	5618.4	5404.6	5305.3	5147.9	5149.2	4820.7	4551.4	4592.3	4503.7	4173.5	4356.4	3961.6	3939.8	3852.8	3535.7
Ischemic heart diseases (I20-I25)	1559.6	1487.4	1428.7	1444.0	1335.4	1359.5	1308.2	1184.5	1215.7	1178.7	1052.3	1054.5	945.4	826.1	758.1	763.9	754.2	898.3	843.2	866.7
Cerebrovascular diseases (I60-I69)	1490.6	1520.4	1458.7	1401.4	1340.6	1273.3	1213.2	1150.5	1085.8	1029.2	982.0	965.5	898.0	839.0	768.8	746.5	675.3	696.1	669.7	627.5
Diseases of arteries, arterioles and capillaries (I70-I79)	1773.3	1704.6	1676.4	1831.3	1662.6	1583.4	1461.4	1545.8	1436.1	1363.5	1245.6	1018.3	1173.0	1317.3	1257.1	1075.2	1059.1	1074.4	1052.8	872.8
Malignant neoplasms (C00-C97) including:	1114.6	1133.2	1130.3	1139.6	1127.4	1131.5	1113.6	1112.6	1099.1	1071.0	1033.9	1004.1	1025.4	997.6	994.3	1081.3	1067.5	1043.0	1061.2	1052.4
Malignant neoplasm of bronchus and lung (C34)	89.4	99.0	99.8	100.9	99.3	100.5	101.5	106.2	103.3	108.8	107.2	105.5	107.4	105.0	115.9	116.1	126.3	124.1	126.4	136.7
Malignant neoplasm of stomach (C16)	85.5	86.1	82.0	78.7	74.9	66.3	66.6	67.2	68.2	58.3	57.8	54.9	52.9	52.5	50.6	51.4	50.6	45.7	45.6	42.7
Colorectal cancer (C18-C20)	152.5	152.1	155.1	156.5	155.7	153.3	150.3	148.0	156.7	157.5	153.1	146.9	153.2	153.7	146.7	158.3	151.1	152.5	153.8	151.5
Malignant neoplasm of breast (C50)	112.1	116.2	111.2	114.7	109.7	117.6	118.3	116.5	117.3	113.4	110.8	109.8	113.8	116.6	116.9	136.6	140.1	146.3	155.4	155.2
Malignant neoplasm of pancreas (C25)	65.4	67.7	67.1	68.1	62.9	63.5	66.5	68.0	71.0	64.2	62.9	59.9	66.9	64.3	65.4	65.2	63.3	62.0	62.5	63.6
Diseases of the respiratory system (J00-J99) including:	526.6	419.0	395.7	450.8	411.7	445.9	412.0	401.6	389.6	408.6	357.3	349.9	362.9	410.0	359.5	438.4	383.4	464.2	466.7	445.5
Chronic obstructive pulmonary disease (J44)	106.9	104.2	97.1	116.0	103.4	108.2	102.8	111.2	107.6	110.2	87.6	88.9	87.8	92.5	76.1	91.1	83.8	88.5	83.0	82.4
Influenza and pneumonia (J09-J18)	379.4	278.8	259.5	295.3	271.9	301.4	272.5	256.0	250.6	259.4	227.3	224.4	248.0	291.0	258.4	308.4	253.8	331.6	347.4	333.1
Diseases of the digestive system (K00-K93) including:	291.7	300.6	310.8	312.2	303.9	305.1	284.2	260.1	252.1	254.8	241.7	235.0	230.7	229.5	205.2	180.4	186.9	192.9	188.1	183.8
Alcoholic liver disease (K70)	30.2	29.2	28.0	24.6	23.1	24.1	20.7	23.4	20.1	20.7	16.9	18.2	17.8	17.8	14.9	12.6	13.7	16.7	15.5	16.6
External causes of mortality (V01-Y98) including:	264.5	254.2	243.1	221.0	210.5	204.1	192.8	171.4	172.1	152.4	147.8	146.5	152.1	146.9	138.2	136.9	129.6	132.6	134.6	131.8
Transport accidents (V01-V99)	21.5	18.6	19.6	17.2	18.5	18.0	15.8	18.6	19.2	15.1	11.8	12.4	11.7	10.9	11.2	10.2	10.0	10.8	10.2	11.9
Falls (W00-W19)	188.9	180.6	159.2	149.2	142.1	128.0	117.8	106.2	106.8	89.8	93.4	94.0	97.6	100.8	94.4	97.4	90.8	90.2	91.6	90.4
Intentional self-harm (X60-X84)	7.4	5.6	8.2	6.2	6.7	5.6	7.4	5.7	5.6	6.1	5.9	5.1	6.2	5.2	4.6	4.4	4.4	3.8	4.6	4.3

Men 65-74

Causes of death	2000	2001	2002	2003	2004	2005	2006	2007	2008	2009	2010	2011	2012	2013	2014	2015	2016	2017	2018	2019
Diseases of the circulatory system (I00-I99) including:	2069.8	1973.9	1836.1	1826.9	1734.3	1664.9	1620.2	1584.3	1539.9	1534.9	1456.6	1363.3	1404.1	1358.5	1271.5	1327.6	1183.5	1140.4	1146.9	1152.0
Ischemic heart diseases (I20-I25)	812.6	758.9	720.9	694.0	650.1	616.4	604.3	585.1	545.4	509.2	500.7	475.8	461.1	414.5	376.5	385.0	361.1	387.6	369.5	388.3
Cerebrovascular diseases (I60-I69)	467.3	459.1	421.4	412.6	391.5	378.4	358.2	341.1	333.3	312.9	299.0	287.1	274.2	258.1	240.7	233.6	225.7	227.9	216.3	211.7
Diseases of arteries, arterioles and capillaries (I70-I79)	257.5	235.5	228.3	258.0	251.9	233.3	220.2	217.8	212.0	213.3	187.5	144.9	156.6	162.5	158.8	148.0	153.6	153.2	154.0	137.7
Malignant neoplasms (C00-C97) including:	1440.1	1431.3	1440.4	1406.4	1405.6	1392.0	1380.1	1374.5	1327.3	1314.7	1285.6	1223.4	1221.7	1195.8	1194.6	1212.8	1183.5	1138.9	1159.1	1106.6
Malignant neoplasm of bronchus and lung (C34)	535.9	531.7	536.7	512.5	513.5	507.0	501.3	492.6	480.1	454.0	448.7	421.9	419.8	411.1	402.6	404.8	406.0	378.1	378.5	357.4
Malignant neoplasm of stomach (C16)	116.5	106.5	111.2	105.4	98.9	99.5	97.7	96.2	89.8	84.9	86.5	82.4	78.4	73.6	74.0	74.8	67.2	64.3	64.3	62.1
Colorectal cancer (C18-C20)	124.3	125.5	130.9	127.1	133.3	132.9	133.6	139.5	147.7	145.9	149.4	143.2	145.5	141.4	141.4	147.2	140.4	138.4	138.5	135.6
Malignant neoplasm of prostate (C61)	93.6	93.1	94.2	82.5	87.6	86.7	86.7	89.5	87.3	93.1	85.4	84.9	83.2	89.4	92.0	94.7	93.8	95.7	99.1	92.1
Malignant neoplasm of pancreas (C25)	51.8	53.1	51.2	55.9	51.4	53.8	51.2	55.6	54.7	57.1	58.4	51.3	57.2	53.6	57.8	55.8	52.3	54.2	53.0	52.9
Diseases of the respiratory system (J00-J99) including:	278.1	242.2	229.4	245.3	233.1	244.4	233.2	241.4	218.5	227.9	205.6	212.0	197.4	216.2	180.9	201.1	196.6	204.6	206.3	213.4
Chronic obstructive pulmonary disease (J44)	171.6	154.0	145.2	160.5	145.5	150.7	145.3	149.5	133.0	134.1	107.0	113.6	97.1	100.3	79.4	83.6	77.9	78.6	75.1	71.2
Influenza and pneumonia (J09-J18)	78.3	63.1	59.3	59.6	64.1	71.2	66.2	70.4	65.4	66.1	69.3	74.0	81.2	97.6	84.7	97.6	92.8	99.4	108.2	122.0
Diseases of the digestive system (K00-K93) including:	160.5	155.5	149.1	151.8	154.6	157.7	140.6	151.9	145.8	147.5	134.1	141.0	136.9	140.7	132.1	118.3	129.5	130.7	136.6	145.0
Alcoholic liver disease (K70)	59.8	53.9	52.3	57.4	56.2	60.8	58.0	63.9	63.1	66.1	56.9	61.7	58.1	57.5	56.5	53.1	57.2	60.5	65.8	69.7
External causes of mortality (V01-Y98) including:	149.7	147.7	149.3	144.2	154.1	148.4	152.1	150.5	151.2	147.2	148.4	144.6	139.6	136.7	129.3	114.5	118.1	115.5	126.0	114.4
Transport accidents (V01-V99)	40.2	35.3	34.2	33.1	35.7	30.4	30.3	30.3	28.9	26.3	23.3	24.8	19.0	19.4	20.3	18.0	16.9	17.6	21.8	18.4
Falls (W00-W19)	25.9	24.8	22.5	22.5	24.9	22.9	23.0	24.0	27.5	22.4	24.4	29.6	30.7	31.1	29.6	26.7	28.6	26.4	26.5	26.0
Intentional self-harm (X60-X84)	29.6	34.7	34.4	33.7	33.8	35.1	34.6	32.2	32.5	40.1	39.6	35.8	34.6	39.1	33.4	30.5	28.6	26.8	26.5	26.7

Men 75+

Causes of death	2000	2001	2002	2003	2004	2005	2006	2007	2008	2009	2010	2011	2012	2013	2014	2015	2016	2017	2018	2019
Diseases of the circulatory system (I00-I99) including:	7928.6	7658.6	7337.0	7520.8	6963.9	6667.3	6449.4	6336.0	6210.0	6304.6	5967.8	5648.6	5737.3	5555.0	5159.0	5304.0	4857.5	4811.6	4651.9	4323.8
Ischemic heart diseases (I20-I25)	2174.9	2080.0	1990.4	2044.5	1875.8	1849.0	1813.4	1658.9	1707.1	1682.3	1534.8	1551.5	1405.5	1236.3	1146.7	1134.2	1138.4	1299.8	1241.8	1252.0
Cerebrovascular diseases (I60-I69)	1659.4	1626.8	1563.7	1539.4	1417.0	1343.7	1303.9	1257.6	1183.9	1147.6	1082.8	1038.1	997.6	903.5	829.2	808.4	759.9	799.3	759.5	696.9
Diseases of arteries, arterioles and capillaries (I70-I79)	2055.1	1938.2	1896.6	2076.5	1908.3	1800.7	1634.8	1740.9	1605.6	1552.7	1454.8	1210.8	1376.9	1543.7	1440.4	1198.7	1174.2	1173.0	1134.0	938.0
Malignant neoplasms (C00-C97) including:	2226.5	2310.1	2364.1	2337.8	2366.3	2316.7	2319.0	2326.5	2336.0	2288.9	2202.3	2192.6	2203.1	2133.5	2104.5	2322.9	2270.0	2201.4	2198.5	2099.3
Malignant neoplasm of bronchus and lung (C34)	520.9	537.3	562.2	551.3	546.0	559.1	551.0	555.3	580.6	558.2	543.8	532.6	529.2	516.2	519.3	525.5	507.4	492.9	494.3	450.3
Malignant neoplasm of stomach (C16)	205.3	200.2	199.2	189.1	189.3	176.0	166.9	169.6	166.4	156.9	155.4	152.9	145.5	146.0	142.5	140.1	137.9	129.0	126.1	117.9
Colorectal cancer (C18-C20)	238.3	240.1	254.1	259.3	255.3	257.2	283.7	283.7	295.0	290.5	295.9	304.3	308.9	301.2	307.7	332.7	331.9	322.9	326.3	316.8
Malignant neoplasm of prostate (C61)	332.2	361.3	373.9	360.4	365.5	355.5	359.0	367.2	348.0	344.4	326.5	325.1	333.5	323.2	318.3	364.4	380.6	378.1	380.6	376.0
Malignant neoplasm of pancreas (C25)	73.9	77.7	81.6	77.8	77.9	73.3	83.1	80.0	86.5	82.6	77.6	76.0	80.9	78.2	81.7	76.5	79.7	72.8	79.8	70.1
Diseases of the respiratory system (J00-J99) including:	1092.1	921.2	920.8	1001.0	966.9	1018.3	974.9	970.6	950.8	964.0	890.5	880.1	883.9	942.0	831.9	937.4	845.7	956.8	972.8	910.5
Chronic obstructive pulmonary disease (J44)	449.4	414.2	441.2	474.3	454.8	471.5	449.9	469.3	463.3	450.6	368.0	381.0	356.1	366.5	295.5	311.2	261.0	294.1	259.9	226.2
Influenza and pneumonia (J09-J18)	564.8	425.3	409.6	451.5	447.5	478.9	463.6	431.7	429.7	440.3	447.9	431.9	473.1	522.5	487.4	561.7	494.7	585.7	645.2	623.6
Diseases of the digestive system (K00-K93) including:	375.0	368.3	380.8	380.0	357.2	368.7	328.5	326.3	307.0	304.9	303.1	286.9	282.2	282.7	245.6	220.0	236.5	235.5	233.4	222.9
Alcoholic liver disease (K70)	59.0	60.2	52.2	46.6	46.4	45.4	41.2	44.2	46.8	40.0	41.1	35.0	37.3	36.1	30.6	28.0	29.8	31.0	32.3	32.2
External causes of mortality (V01-Y98) including:	338.1	323.3	327.9	305.6	283.4	291.8	283.3	277.0	277.9	249.4	248.6	244.3	257.0	241.8	238.9	225.9	217.6	217.4	221.3	209.9
Transport accidents (V01-V99)	62.9	45.0	51.8	45.7	48.4	43.2	38.1	44.4	43.9	35.3	28.0	27.7	26.9	25.0	26.8	23.9	24.9	23.0	27.3	26.4
Falls (W00-W19)	160.6	165.0	154.5	143.6	127.4	128.4	127.2	119.3	119.9	107.6	115.2	112.4	121.6	125.2	125.0	118.1	105.4	113.5	110.2	110.6
Intentional self-harm (X60-X84)	41.7	30.5	34.4	32.0	32.5	33.9	35.1	34.5	35.7	36.6	34.9	34.6	35.8	35.5	31.9	33.7	31.3	25.4	27.3	31.8
