# Supplementary material for: Changes in mortality of Polish residents in the early and late old age due to main causes of death from 2000 to 2019
Source: Front Public Health. 2023 Mar 6;11:1060028. doi: 10.3389/fpubh.2023.1060028 (PMC10025537; doi:10.3389/fpubh.2023.1060028)
Supplement: Supplementary file 2 [file Table_2.pdf]

Supplement 2. SDR time trends due to main causes of mortality in Poland in 2000-2019 – joinpoint regression analysis

**Women**	**Number of joinpoints**	**Years**	**APC (95% CI)**	**AAPC (95% CI)**
**Age 65-74**				
Diseases of circulatory system (I00-I99) including:	1	2000-2006	-5.6* (-6.8; -4.4)	-4.1* (-4.5; -3.6)
		2006-2019	-3.3* (-3.7; -3.0)	
Ischemic heart diseases (I20-I25)	1	2000-2015	-6.0* (-6.4; -5.6)	-4.4* (-5.0; -3.7)
		2015-2019	2.1 (-0.9; 5.2)	
Cerebrovascular diseases (I60-I69)	3	2000-2008	-6.5* (-6.9; -6.0)	-5.6* (-6.4; -4.8)
		2008-2012	-4.4* (-6.6; -2.1)	
		2012-2015	-8.3* (-12.5; -3.9)	
		2015-2019	-3.0* (-4.4; -1.6)	
Diseases of arteries, arterioles and capillaries (I70-I79)	0	2000-2019	-4.2* (-4.9; -3.5)	
Malignant neoplasms (C00-C97) including:	1	2000-2006	-1.0* (-1.7; -0.3)	0.0 (-0.2; 0.3)
		2006-2019	0.5*(0.3; 0.7)	
Malignant neoplasms of bronchus and lung (C34)	1	2000-2005	1.5 (-0.7; 3.8)	4.1* (3.4; 4.7)
		2005-2019	5.0* (4.5; 5.5)	
Malignant neoplasms of stomach (C16)	1	2000-2002	-7.8 (-21.3; 8.1)	-3.2* (-4.7; -1.6)
		2002-2019	-2.6* (-3.2; -21.)	
Colorectal cancer (C18-C20)	0	2000-2019	-0.3 (-0.7; 0.0)	
Malignant neoplasms of breast (C50)	2	2000-2010	0.3 (-0.2; 0.8)	0.6 (0.0; 1.3)
		2010-2014	3.0 (0.0; 6.2)	
		2014-2019	-0.5 (-1.8; 0.9)	
Malignant neoplasms of pancreas (C25)	0	2000-2019	0.7* (0.3; 1.0)	
Diseases of respiratory system (J00-J99) including:	1	2000-2002	-14.7 (-30.3; 4.3)	0.1 (-1.9; 2.2)
		2002-2019	2.0* (1.3; 2.8)	
Chronic obstructive pulmonary disease (J44)	0	2000-2019	0.1 (-0.5; 0.7)	
Influenza and pneumonia (J09-J18)	1	2000-2008	-3.1* (-6.1; -0.1)	2.3* (0.7; 3.9)

		2008-2019	6.4* (4.4; 8.4)	
Diseases of the digestive system (K00-K93) including:	1	2000-2015	-2.5* (-2.9; -2.1)	-1.5* (-2.1; -0.8)
		2015-2019	2.5 (-0.5; 5.7)	
Alcoholic liver disease (K70)	3	2000-2003	-9.9* (-16.4; -2.8)	0.2 (-2.4; 2.8)
		2003-2006	7.1 (-7.8; 24.5)	
		2006-2016	-1.5* (-2.9; -0.2)	
		2016-2019	10.2* (2.2; 18.8)	
External causes of mortality (V01-Y98) including:	0	2000-2019	-2.9* (-3.2; -2.6)	
Transport accidents (V01-V99)	2	2000-2008	-2.2* (-4.4; 0.0)	-3.9* (-6.9; -0.9)
		2008-2011	-12.6 (-28.8; 7.3)	
		2011-2019	-2.2 (-4.3; 0.0)	
Falls (W00-W19)	1	2000-2007	-7.1* (-10.8; -3.3)	-2.2* (-3.9; -0.5)
		2007-2019	0.7 (-1.0; 2.6)	
Intentional self-harm (X60-X84)	1	2000-2009	0.2 (-2.1; 2.6)	-2.8* (-4.2; -1.5)
		2009-2019	-5.5* (-7.4; -3.6)	
**Age 75+**				
Diseases of circulatory system (I00-I99) including:	0	2000-2019	-3.0* (-3.2; -2.8)	
Ischemic heart diseases (I20-I25)	2	2000-2011	-3.5* (-4.3; -2.6)	-3.0* (-4.8; -1.1)
		2011-2014	-11.2 (-21.6; 0.6)	
		2014-2019	3.5* (0.6; 6.4)	
Cerebrovascular diseases (I60-I69)	0	2000-2019	-4.9* (-5.1; -4.6)	
Diseases of arteries, arterioles and capillaries (I70-I79)	0	2000-2019	-3.4* (-4.0; -2.8)	
Malignant neoplasms (C00-C97) including:	2	2000-2007	-0.1 (-0.9; 0.8)	-0.3 (-1.0; 0.4)
		2007-2011	-2.7 (-5.7; 0.4)	
		2011-2019	0.7* (0.0; 1.4)	
Malignant neoplasms of bronchus and lung (C34)	1	2000-2013	1.1* (0.6; 1.5)	1.8* (1.3; 2.3)
		2013-2019	3.5* (2.0; 5.0)	
Malignant neoplasms of stomach (C16)	0	2000-2019	-3.6* (-3.9; -3.3)	
Colorectal cancer (C18-C20)	0	2000-2019	-0.1 (-0.2; 0.1)	
Malignant neoplasms of breast (C50)	1	2000-2012	-0.1 (-0.7; 0.4)	1.8* (1.2; 2.3)
		2012-2019	5.1* (3.8; 6.5)	

Malignant neoplasms of pancreas (C25)	0	2000-2019	-0.3* (-0.6; 0.0)	
Diseases of respiratory system (J00-J99) including:	1	2000-2011	-2.3* (-3.8; -0.8)	-0.1 (-1.3; 1.2)
		2011-2019	3.1* (0.6; 5.7)	
Chronic obstructive pulmonary disease (J44)	0	2000-2019	-1.6* (-2.2; -1.0)	
Influenza and pneumonia (J09-J18)	1	2000-2011	-2.7* (-4.6; -0.9)	0.5 (-1.1; 2.1)
		2011-2019	5.1* (1.8; 8.4)	
Diseases of the digestive system (K00-K93) including:	1	2000-2003	2.2 (-4.3; 9.2)	-2.7* (-3.7; -1.7)
		2003-2019	-3.6* (-4.1; -3.1)	
Alcoholic liver disease (K70)	1	2000-2015	-4.8* (-5.6; -4.1)	-3.1* (-4.4; -1.8)
		2015-2019	3.7 (-2.5; 10.3)	
External causes of mortality (V01-Y98) including:	1	2000-2010	-5.7* (-6.3; -5.1)	-3.7* (-4.2; -3.3)
		2010-2019	-1.5* (-2.3; -0.8)	
Transport accidents (V01-V99)	2	2000-2008	-1.6 (-3.9; 0.8)	-3.2 (-6.4; 0.1)
		2008-2011	-13.3 (-30.5; 8.1)	
		2011-2019	-0.8 (-3.1; 1.7)	
Falls (W00-W19)	1	2000-2009	-7.4* (-8.2; -6.6)	-3.7* (-4.2; -3.2)
		2009-2019	-0.3 (-1.0; 0.4)	
Intentional self-harm (X60-X84)	0	2000-2019	-2.8* (-3.7; -1.9)	

* p<0,05

Men	Number of joinpoints	Years	APC (95% CI)	AAPC (95% CI)
**Age 65-74**				
Diseases of circulatory system (I00-I99) including:	0		-3.0* (-3.2; -2.8)	
Ischemic heart diseases (I20-I25)	1	2000-2016	-4.8* (-5.1; -4.5)	-3.8* (-4.4; -3.2)
		2016-2019	2.0 (-1.9; 6.2)	
Cerebrovascular diseases (I60-I69)	1	2000-2015	-4.6* (-4.7; -4.4)	-4.1* (-4.4; -3.9)
		2015-2019	-2.6* (-3.9; -1.2)	
Diseases of arteries, arterioles and capillaries (I70-I79)	2	2000-2008	-1.5 (-3.1; 0.1)	-2.8* (-5.0; -0.6)
		2008-2011	-10.4 (-22.8; 3.9)	
		2011-2019	-1.1 (-2.6; 0.6)	
Malignant neoplasms (C00-C97) including:	2	2000-2007	-0.7* (-1.2; -0.2)	-1.3* (-1.7; -0.9)
		2007-2011	-2.5* (-4.4; -0.6)	
		2011-2019	-1.2* (-1.6; -0.7)	
Malignant neoplasms of bronchus and lung (C34)	3	2000-2007	-1.2* (-1.8; -0.6)	-2.1* (-2.7; -1.5)
		2007-2011	-3.8* (-5.9; -1.7)	
		2011-2016	-1.2 (-2.6; 0.2)	
		2016-2019	-3.5* (-5.6; -1.3)	
Malignant neoplasms of stomach (C16)	0	2000-2019	-3.2* (-3.4; -3.0)	
Colorectal cancer (C18-C20)	1	2000-2010	1.8* (1.3; 2.3)	0.5* (0.2; 0.9)
		2010-2019	-0.8* (-1.4; -0.3)	
Malignant neoplasms of prostate (C61)	1	2000-2011	-0.6 (-1.4; 0.3)	0.4 (-0.3; 1.0)
		2011-2019	1.6* (0.2; 3.0)	
Malignant neoplasms of pancreas (C25)	0	2000-2019	0.2 (-0.2; 0.5)	
Diseases of respiratory system (J00-J99) including:	0	2000-2019	-1.4* (-1.9; -0.9)	
Chronic obstructive pulmonary disease (J44)	1	2000-2007	-2.0 (-4.5; 0.5)	-4.6* (-5.6; -3.5)
		2007-2019	-6.0* (-7.1; -4.9)	
Influenza and pneumonia (J09-J18)	1	2000-2002	-14.2 (-32.1; 8.4)	1.9 (-0.5; 4.3)
		2002-2019	4.0* (3.1; 4.8)	
Diseases of the digestive system (K00-K93) including:	1	2000-2016	-1.4* (-1.9; -0.9)	-0.6 (-1.6; 0.4)

		2016-2019	3.8 (-2.5; 10.5)	
Alcoholic liver disease (K70)	3	2000-2002	-4.8 (-16.1; 8.1)	0.9 (-0.8; 2.6)
		2002-2008	3.3* (0.4; 6.2)	
		2008-2015	-2.5* (-4.6; -0.4)	
		2015-2019	6.6* (2.4; 10.9)	
External causes of mortality (V01-Y98) including:	1	2000-2009	0.1 (-1.0; 1.2)	-1.5* (-2.1; -0.8)
		2009-2019	-2.9* (-3.8; -2.0)	
Transport accidents (V01-V99)	1	2000-2016	-5.0* (-5.9; -4.2)	-3.6* (-5.4; -1.9)
		2016-2019	4.2 (-7.3; 17.1)	
Falls (W00-W19)	2	2000-2009	-0.3 (-2.0; 1.4)	0.3 (-2.6; 3.2)
		2009-2012	9.5 (-9.4; 32.3)	
		2012-2019	-2.7* (-5.1; -0.2)	
Intentional self-harm (X60-X84)	1	2000-2012	1.2* (0.0; 2.4)	-1.3* (-2.4; -0.2)
		2012-2019	-5.5* (-8.0; -2.9)	
**Age 75+**				
Diseases of circulatory system (I00-I99) including:	0	2000-2019	-2.9* (-3.1; -2.7)	
Ischemic heart diseases (I20-I25)	2	2000-2011	-3.0* (-3.7; -2.4)	-2.7* (-4.3; -1.2)
		2011-2014	-9.7* (-18.5; 0.0)	
		2014-2019	2.5* (0.2; 4.8)	
Cerebrovascular diseases (I60-I69)	0	2000-2019	-4.7* (-4.9; -4.4)	
Diseases of arteries, arterioles and capillaries (I70-I79)	0	2000-2019	-3.6* (-4.2; -3.0)	
Malignant neoplasms (C00-C97) including:	0	2000-2019	-0.4* (-0.7; -0.2)	
Malignant neoplasms of bronchus and lung (C34)	1	2000-2008	0.8* (0.0; 1.6)	-0.6* (-1.0; -0.2)
		2008-2019	-1.6* (-2.1; -1.2)	
Malignant neoplasms of stomach (C16)	0	2000-2019	-2.7* (-2.9; -2.5)	
Colorectal cancer (C18-C20)	1	2000-2016	2.0* (1.7; 2.3)	1.5* (0.8; 2.1)
		2016-2019	-1.5 (-5.3; 2.6)	
Malignant neoplasms of prostate (C61)	3	2000-2002	6.0 (-1.7; 14.3)	0.6 (-0.7; 2.0)
		2002-2013	-1.5* (-2.0; -0.9)	
		2013-2016	5.7 (-2.0; 14.0)	
		2016-2019	0.0 (-3.7; 3.8)	

Malignant neoplasms of pancreas (C25)	0	2000-2019	-0.1 (-0.6; 0.3)	
Diseases of respiratory system (J00-J99) including:	0	2000-2019	-0.5* (-1.0; -0.1)	
Chronic obstructive pulmonary disease (J44)	1	2000-2007	1.3 (-1.1; 3.8)	-3.1* (-4.1; -2.1)
		2007-2019	-5.6* (-6.6; -4.6)	
Influenza and pneumonia (J09-J18)	1	2000-2010	-0.9 (-2.7; 0.9)	1.5* (0.2; 2.9)
		2010-2019	4.3* (2.1; 6.6)	
Diseases of the digestive system (K00-K93) including:	0	2000-2019	-3.1* (-3.5; -2.7)	
Alcoholic liver disease (K70)	1	2000-2016	-4.1* (-4.8; -3.3)	-2.9* (-4.5; -1.3)
		2016-2019	3.3 (-7.0; 14.7)	
External causes of mortality (V01-Y98) including:	0	2000-2019	-2.4* (-2.6; -2.2)	
Transport accidents (V01-V99)	1	2000-2015	-5.8* (-7.0; -4.5)	-4.0* (-6.1; -1.9)
		2015-2019	2.9 (-6.8; 13.8)	
Falls (W00-W19)	2	2000-2009	-4.4* (-5.6; -3.2)	-2.2* (-3.7; -0.7)
		2009-2013	3.1 (-3.7; 10.4)	
		2013-2019	-2.3* (-4.6; 0.0)	
Intentional self-harm (X60-X84)	0	2000-2019	-0.9* (-1.6; -0.1)	

* p<0,05
